# A nomogram combining long non-coding RNA expression profiles and clinical factors predicts survival in patients with bladder cancer

**DOI:** 10.18632/aging.102782

**Published:** 2020-02-12

**Authors:** Yifan Wang, Lutao Du, Xuemei Yang, Juan Li, Peilong Li, Yinghui Zhao, Weili Duan, Yingjie Chen, Yunshan Wang, Haiting Mao, Chuanxin Wang

**Affiliations:** 1 Department of Clinical Laboratory, The Second Hospital of Shandong University, Jinan, Shandong, China; 2 Tumor Marker Detection Engineering Technology Research Center of Shandong Province, Jinan, Shandong, China; 3 Tumor Marker Detection Engineering Laboratory of Shandong Province, Jinan, Shandong, China; 4 The Clinical Research Center of Shandong Province for Clinical Laboratory, Jinan, Shandong, China

**Keywords:** bladder cancer, long non-coding RNA, survival, score system, nomogram

## Abstract

Bladder cancer (BCa) is a heterogeneous disease with various tumorigenic mechanisms and clinical behaviors. The current tumor-node-metastasis (TNM) staging system is inadequate to predict overall survival (OS) in BCa patients. We developed a BCa-specific, long-non-coding-RNA (lncRNA)-based nomogram to improve survival prediction in BCa. We obtained the large-scale gene expression profiles of samples from 414 BCa patients in The Cancer Genome Atlas database. Using an lncRNA-mining computational framework, we identified three OS-related lncRNAs among 826 lncRNAs that were differentially expressed between BCa and normal samples. We then constructed a three-lncRNA signature, which efficiently distinguished high-risk from low-risk patients and was even viable in the TNM stage-II, TNM stage-III and ≥65-year-old subgroups (all *P*<0.05). Using clinical risk factors, we developed a signature-based nomogram, which performed better than the molecular signature or clinical factors alone for prognostic prediction. A bioinformatical analysis revealed that the three OS-related lncRNAs were co-expressed with genes involved in extracellular matrix organization. Functional assays demonstrated that RNF144A-AS1, one of the three OS-related lncRNAs, promoted BCa cell migration and invasion *in vitro*. Our three-lncRNA signature-based nomogram effectively predicts the prognosis of BCa patients, and could potentially be used for individualized management of such patients.

## INTRODUCTION

Bladder cancer (BCa) is the 10th most common cancer worldwide, accounting for an estimated 549,393 newly diagnosed cases and 199,922 deaths in 2018. A strong male predominance has been observed, with four-fifths of all BCa patients being men [1–3]. Of newly diagnosed BCa cases, nearly 75% present as non-muscle-invasive bladder cancer, which is confined to the muscularis propria. In spite of endoscopic and intravesical treatments, more than half of cases recur or progress to aggressive muscle-invasive bladder cancer [4–8]. With the progression of BCa, the five-year survival rate gradually declines, falling to less than 50% at later stages (*i.e.*, muscle invasive and beyond) [9, 10]. Thus, the early assessment of individual outcomes is imperative.

Clinicopathological factors such as the tumor-node-metastasis (TNM) stage and lymph node status have been used most frequently to assess BCa outcomes in clinical practice. The overall survival (OS) is worse in patients with higher-stage or lymph-node-positive BCa [11, 12]. However, the prognostic determination is often based on inherent anatomical information alone, so it is difficult to predict disease progression due to the biological heterogeneity of BCa [5]. Thus, there is an urgent need to identify reliable biomarkers to predict the prognosis and guide the treatment of patients with BCa.

Genome-wide sequencing has revealed the extensive landscape of the mammalian genome, including non-protein-coding regions that are transcribed into RNA. ‘Long non-coding RNA’ (lncRNA) refers to any polyadenylated RNA >200 bp long that does not appear to encode a protein [13, 14]. By binding to cellular nucleic acids, proteins and other macromolecules, lncRNAs exert elaborate regulatory effects that can ultimately drive tumorigenesis and metastasis [15–19]. LncRNAs thus comprise an enormous reservoir of potential cancer treatment targets, and have been found to mark specific states of tumor progression and even predict outcomes [20–25]. Although some molecular biomarkers have been identified and tested among BCa patients [26–28], most studies have had small sample sizes, employed different platforms or failed to combine diverse prognostic variables. For these reasons, the identification of robust prognostic biomarkers remains an urgent clinical challenge.

The Cancer Genome Atlas (TCGA, http://cancergenome.nih.gov/) consortium has been characterizing the genomic landscape through high-throughput molecular profiling analyses of large available cohorts, which has greatly facilitated the discovery of cancer-specific biomarkers [29–33]. Herein, we used a rigorous computational framework to mine lncRNA expression profiles and clinical data from the Bladder Urothelial Carcinoma Project of TCGA (‘TCGA-BLCA Project’). We then constructed a three-lncRNA signature-based nomogram to predict the OS of patients with BCa.

## RESULTS

### Candidate OS-related lncRNAs from BCa patients

**Figure 1:**
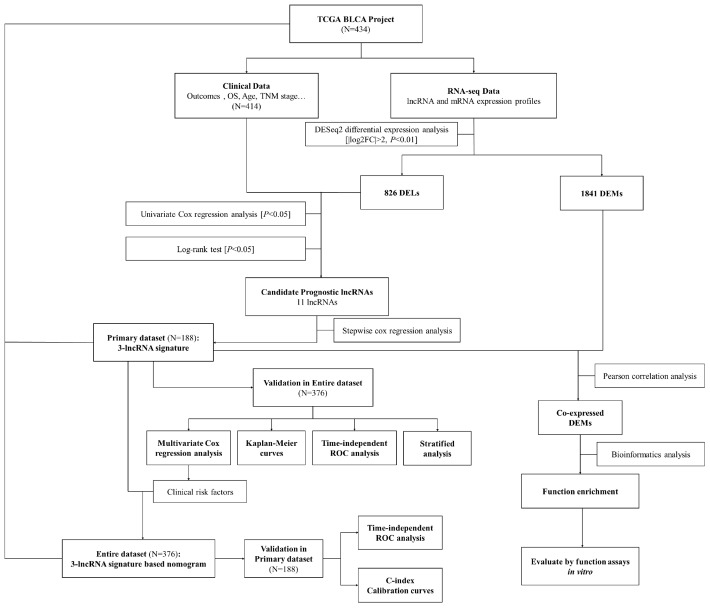
**Flowchart of this study.**

**Figure 2:**
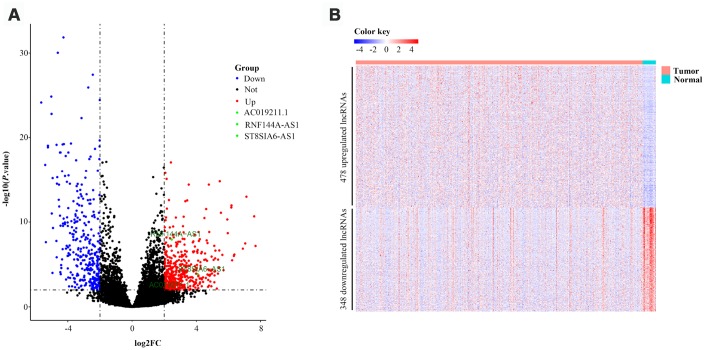
**Volcano plot and heatmap of 826 lncRNAs in bladder cancer patients from TCGA-BLCA Project.** (**A**) Volcano plot of 826 lncRNAs in bladder cancer samples from TCGA-BLCA Project. Green points represent candidate OS-related lncRNAs. (**B**) Heatmap of 826 lncRNAs in bladder cancer samples from TCGA-BLCA Project. Blue and red indicate downregulated and upregulated lncRNAs, respectively.

The overall design and flowchart of this study is presented in Figure 1. In total, 414 BCa patients from TCGA database were included. We compared the lncRNA and mRNA expression profiles of the 414 BCa samples with those of 19 normal samples. We identified 826 differentially expressed lncRNAs (DELs) and 1841 differentially expressed mRNAs (DEMs) with a log_2_|fold change| >2 and an adjusted *P* value <0.01. Of the 826 DELs, 478 lncRNAs were found to be upregulated and 348 were found to be downregulated in the BCa patients. The volcano plots and heatmaps of the DELs and DEMs were visualized with the “ggplot2” and “pheatmap” packages of R software, and are shown in Figure 2 and Supplementary Figure 1, respectively.

After the exclusion of four patients with insufficient survival data, 410 BCa patients remained in our study. All 826 DELs were subjected to univariate Cox proportional hazards regression (CPHR) analysis and Kaplan-Meier analysis, with OS as the dependent variable and the lncRNA level as the explanatory variable. As shown in Supplementary Table 1, 11 lncRNAs were significantly associated with the OS of BCa patients (all *P*<0.05). Ten of these 11 lncRNAs (AC007406.3, AC019211.1, AC022613.1, AC112721.1, AL391704.1, LINC01602, ST8SIA6-AS1, LINC01929, LINC01971 and RNF144A-AS1) had hazard ratios (HRs) greater than 1, suggesting that their overexpression was associated with shorter OS. On the other hand, the HR for SMC2-AS1 was less than 1, with the opposite implications. The Kaplan-Meier analysis curves were consistent with the univariate CPHR analysis results (Supplementary Figure 2). Thus, we considered these dysregulated lncRNAs as candidate OS-related lncRNAs.

**Table 1:** Baseline clinical characteristics of 376 bladder cancer cases involved in this study.

**Characteristic**	**Primary dataset**	**Entire dataset**	***P* Value**
	**n=188**	**n=376**	
**Age (years)**			0.706
≥65	126 (67.02%)	246 (65.43%)	
<65	62 (32.98%)	130 (34.57%)	
Gender			0.946
Female	49 (26.06%)	99 (26.33%)	
Male	139 (73.94%)	277 (73.67%)	
**TNM stage**			0.688
I-II	49 (26.06%)	104 (27.66%)	
III-IV	139 (73.94%)	272 (72.34%)	
**Tumor stage**			0.700
T0-T2	57 (30.32%)	120 (31.91%)	
T3-T4	131 (69.68%)	256 (68.09%)	
**Lymph node metastasis**			0.899
Nx	13 (6.91%)	28 (7.45%)	
no	108 (57.45%)	221 (58.78%)	
yes	67 (35.64%)	127 (33.78%)	
**Distant metastasis**			0.937
Mx	90 (47.87%)	186 (49.47%)	
no	94 (50.00%)	182 (48.40%)	
yes	4 (2.13%)	8 (2.13%)	

### Identification and validation of a three-lncRNA signature for survival prediction

We further reduced the BCa dataset based on the availability of clinical data, and thus excluded 34 patients without data on clinical characteristics such as the TNM stage and age. Of the remaining 376 BCa patients, 188 were randomly designated as the ‘primary dataset’, while the complete group of 376 patients was enrolled as the ‘entire dataset’. The clinical characteristics did not differ significantly between the two datasets (all *P*>0.05). The detailed characteristics are listed in Table 1.

To identify the best-fit OS-related lncRNAs, we filtered these candidate lncRNAs through a multivariate CPHR analysis (stepwise model). We used the Akaike information criterion (AIC) to avoid over-fitting. The three OS-related lncRNAs with the largest likelihood ratios and lowest AIC values (RNF144A-AS1, AC019211.1 and ST8SIA6-AS1) were selected from the stepwise model (Table 2) and integrated into a predictive signature based on their risk coefficients. The formula was as follows: Risk Score = (0.228 × Expression_RNF144A-AS1_) + (0.436 × Expression_AC019211.1_) + (0.116 × Expression_ST8SIA6-AS1_).

**Table 2:** Three lncRNAs significantly associated with overall survival in the primary dataset.

**Gene name**	**Coefficient**	**Type**	**Down/up-regulated**	**HR**	**95%CI**	***P* value**
RNF144A-AS1	0.228	Risky	Up	1.256	1.065-1.480	0.007
AC019211.1	0.436	Risky	Up	1.547	1.181-2.026	0.002
ST8SIA6-AS1	0.116	Risky	Up	1.123	1.022-1.235	0.016

Abbreviations: HR, hazard ratio; CI, confidence interval.

**Figure 3:**
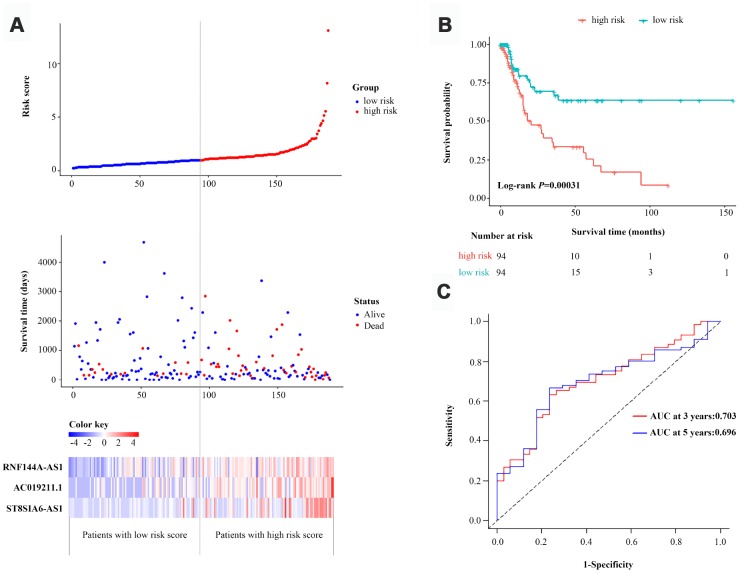
**Identification and assessment of a three-lncRNA signature to predict OS in the primary dataset.** (**A**) The risk score distribution, OS status and heatmap of the three-lncRNA signature in the primary dataset. (**B**) Kaplan-Meier curves for OS based on the three-lncRNA signature in the primary dataset. The tick-marks on the curve represent the censored subjects. The number of patients at risk is listed below the curve. (**C**) Time-dependent ROC curve analysis of the three-lncRNA signature for predicting OS in the primary dataset.

Then, we calculated the three-lncRNA-based risk score for each BCa patient in the primary dataset. Using the median risk score as the cut-off value, we classified the 188 patients into a high-risk group (n=94) and a low-risk group (n=94). The distributions of the lncRNA-based risk scores, OS statuses and three lncRNA expression profiles in the primary dataset are shown in Figure 3A. The heatmap revealed that all three of the high-risk lncRNAs were expressed at higher levels in the high-risk group than in the low-risk group. Kaplan-Meier curve analysis clearly demonstrated that the high-risk group had a poorer prognosis than the low-risk group (*P*=3.1E-04, log-rank test) (Figure 3B). Subsequently, we constructed a time-dependent receiver operating characteristic (ROC) curve with the primary dataset. As shown in Figure 3C, the area under the time-dependent ROC curve (AUC) of the three-lncRNA signature reached 0.703 (95% confidence interval [CI]=0.593-0.814) at three years and 0.696 (95% CI=0.563-0.829) at five years.

**Figure 4:**
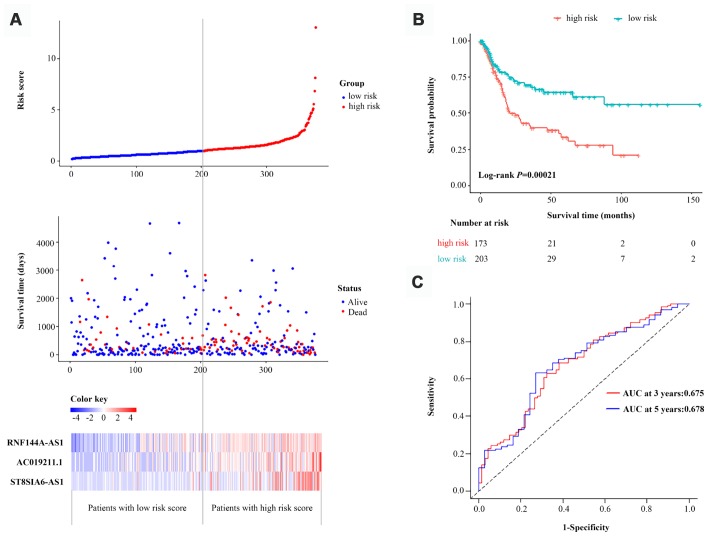
**Validation of the three-lncRNA signature in the entire dataset.** (**A**) The risk score distribution, OS status and heatmap of the three-lncRNA signature in the entire dataset. (**B**) Kaplan-Meier curves for the OS of bladder cancer patients based on the three-lncRNA signature in the entire dataset. The tick-marks on the curve represent the censored subjects. The number of patients at risk is listed below the curve. (**C**) Time-dependent ROC curve depicting the predictive accuracy of the signature for OS in the entire dataset.

The performance of the three-lncRNA signature for predicting survival was then validated with the entire dataset (n=376). When we used the three-lncRNA signature and cut-off value derived from the primary dataset, the distributions of the three-lncRNA-based risk scores, OS statuses and three lncRNA expression profiles in the entire dataset were consistent with the findings described above (Figure 4A). Similar to the results in the primary dataset, a Kaplan-Meier curve analysis indicated that the survival time of BCa patients was significantly shorter in the high-risk group (n=173) than in the low-risk group (n=203) (*P*=2.1E-04, log-rank test) (Figure 4B). The AUC of the three-lncRNA signature was 0.675 (95% CI=0.593-0.759) at three years and 0.678 (95% CI=0.576-0.781) at five years in the entire dataset (Figure 4C). Thus, the predictive performance of the three-lncRNA signature for BCa patients was great in both the primary dataset and the entire dataset.

### The prognostic value of the three-lncRNA signature was independent from those of conventional clinical risk factors

Next, we tested whether the prognostic performance of the three-lncRNA signature was independent from those of conventional clinical risk factors. A multivariate CPHR analysis demonstrated that the HR of a high *vs.* low risk score was 2.368 (*P*=0.003, 95% CI=1.345-4.168) in the primary dataset and 1.856 (*P*=0.002, 95% CI=1.243–2.770) in the entire dataset (Table 3 and Supplementary Table 2), indicating that the three-lncRNA signature could independently predict the prognoses of BCa patients.

**Table 3:** Univariate and multivariate Cox proportional hazards regression analysis of 3-lncRNA signature and clinical risk factors in the entire dataset.

**Characteristic**	**Univariate analysis**		**Multivariate analysis**	
	**HR (95%CI)**	***P*-Value**	**HR (95%CI)**	***P* Value**
Age (≥65 *vs.* <65)	1.585 (1.023-2.456)	0.039	1.025 (1.005-1.047)	0.016
Gender (male *vs.* female)	0.800 (0.532-1.205)	0.286		
TNM stage (III-IV *vs.* I-II)	4.249 (2.143-8.424)	< 0.001	3.900 (1.962-7.752)	< 0.001
Tumor stage (T3-T4 *vs.* T0-T2)	2.720 (1.616-4.577)	< 0.001		
Lymph node metastasis (yes *vs.* no)	2.455 (1.639-3.676)	< 0.001		
Distant metastasis (yes *vs.* no)	2.321 (0.712-7.568)	0.163		
Risk score (high *vs.* low)	2.088 (1.403-3.108)	< 0.001	1.856 (1.243-2.770)	0.002

Notes: Bold values indicate statistical significance (*P*<0.05).

Abbreviations: HR, hazard ratio; CI, confidence interval.

**Figure 5:**
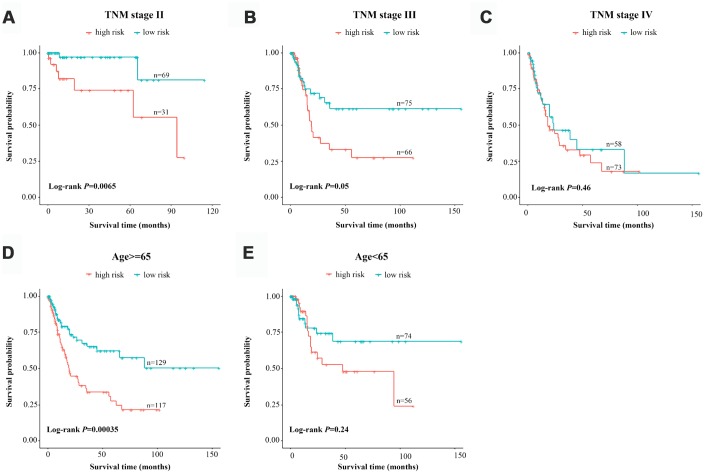
**Risk-stratified analysis of the three-lncRNA signature for bladder cancer patients.** Kaplan-Meier analysis of patients in the stage-II subgroup (**A**), stage-III subgroup (**B**), stage-IV subgroup (**C**), ≥65-year-old subgroup (**D**) and <65-year-old subgroup (**E**). The tick-marks on the curve represent the censored subjects. The differences between the two risk groups were assessed with two-sided log-rank tests.

Considering the number of BCa patients, we performed a risk-stratified analysis with the entire dataset. The 376 BCa patients were stratified into a stage-I subgroup (n=4), stage-II subgroup (n=100), stage-III subgroup (n=141) and stage-IV subgroup (n=131) based on their TNM stage. Except for the stage-I subgroup, which had a small sample size, each subgroup was divided into a high-risk group and a low-risk group based on the risk scores proposed above. We found that the classification efficiency of the three-lncRNA signature was limited when it was applied to certain subgroups. As shown in the Kaplan-Meier curves, for the stage-II and stage-III subgroups, patients in the high-risk group had significantly poorer survival than those in the low-risk group (stage-II subgroup, *P*=0.0065; stage-III subgroup, *P*=0.05, log-rank test) (Figure 5A and 5B). However, the three-lncRNA signature did not reach the threshold of significance in the stage-IV subgroup (Figure 5C). When a stratified analysis was carried out based on age, only in the ≥65-year-old subgroup did the three-lncRNA signature subdivide patients into a high-risk group and a low-risk group with significantly different survival (*P*=3.5E-04, log-rank test) (Figure 5D and 5E). Thus, although the three-lncRNA signature could be viewed as an independent prognostic predictor for BCa patients, its performance was limited to specific subgroups.

### Development of a nomogram combining the three-lncRNA signature with clinical risk factors

Clinical risk factors such as the TNM stage and age are still vital predictors of OS in BCa patients. Therefore, we integrated these traditional risk factors with our three-lncRNA signature to develop an efficient quantitative method of predicting OS. To prevent valuable variables from being overlooked due to the smaller sample size of the primary dataset, we first evaluated the prognostic value of several clinical risk factors in univariate and multivariate CPHR analyses of the entire dataset. We found that, in addition to the three-lncRNA signature, age (≥65 *vs.* <65) and TNM stage (III-IV *vs.* I-II) were significantly associated with OS (all *P*<0.05) (Table 3). We excluded the tumor stage, lymph node metastasis and distant metastasis from the multivariate CPHR analysis because these factors correlate closely with the TNM stage and thus could have caused spurious associations and unreliable effect estimates.

**Figure 6:**
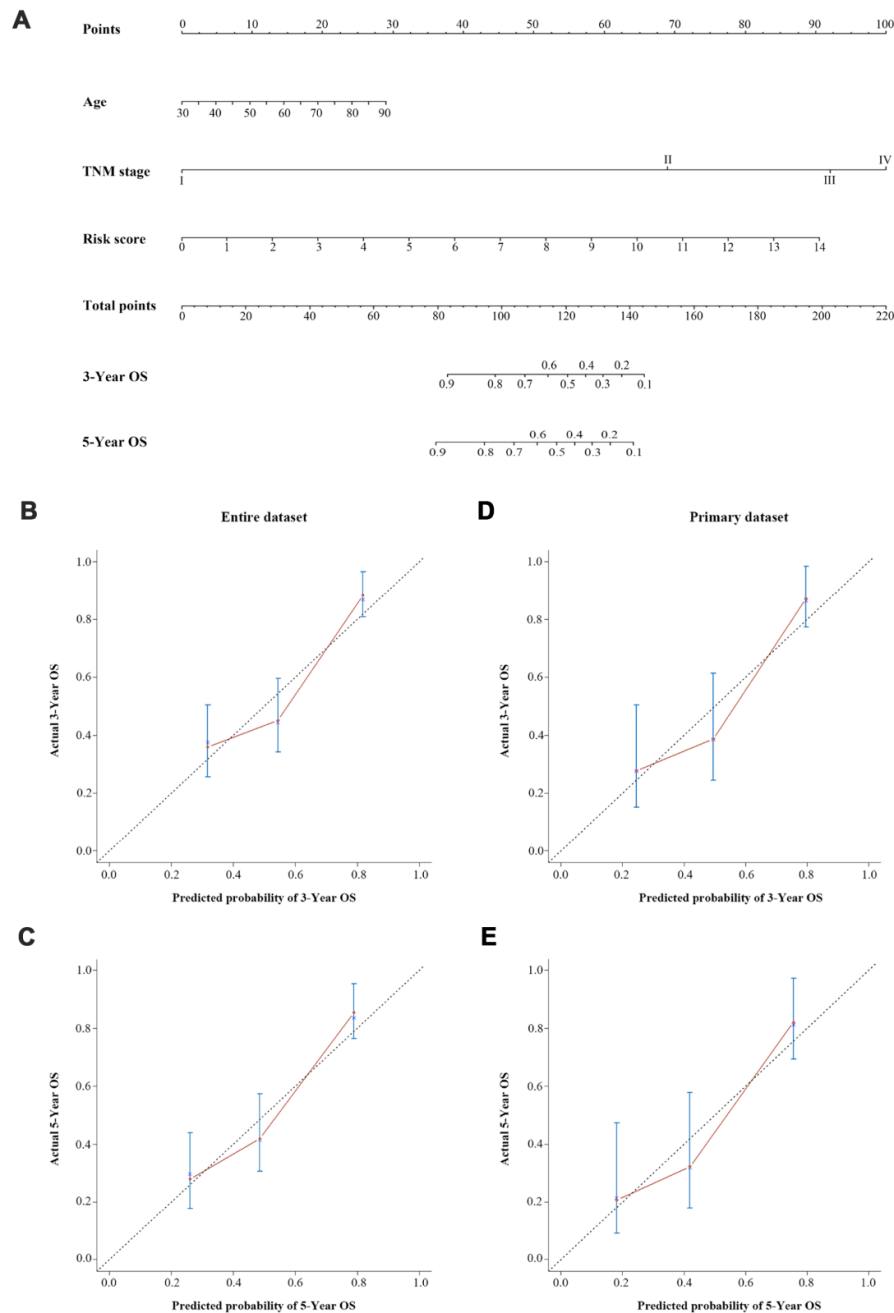
**A three-lncRNA signature-based nomogram to predict three- and five-year OS in bladder cancer patients.** (**A**) Nomogram for predicting OS. Instructions: Locate each characteristic on the corresponding variable axis, and draw a vertical line upwards to the points axis to determine the specific point value. Repeat this process. Tally up the total points value and locate it on the total points axis. Draw a vertical line down to the three- or five-year OS to obtain the survival probability for a specific bladder cancer patient. (**B**–**E**) Calibration plots of the nomogram for predicting OS at three years (**B**) and five years (**C**) in the entire dataset, and at three years (**D**) and five years (**E**) in the primary dataset. The 45-degree dotted line represents a perfect prediction, and the red lines represent the predictive performance of the nomogram.

Ultimately, on the basis of clinical judgment and statistical significance, we developed a three-lncRNA signature-based nomogram, which integrated the three-lncRNA signature and two clinical risk factors (age and TNM stage). We then used this nomogram to predict the three-year and five-year survival of BCa patients (Figure 6A). As shown in the nomogram, the TNM stage contributed the most to the three- and five-year OS, followed closely by the three-lncRNA signature and age. This user-friendly graphical tool allowed us to determine the three- and five-year OS probability for each BCa patient easily.

We then evaluated the discrimination and calibration abilities of the prognostic nomogram by using a concordance index (C-index) and calibration plots. An internal validation using a bootstrap with 1000 resamplings revealed that the nomogram performed well for discrimination: the C-index was 0.688 (95% CI=0.631-0.745) for the entire dataset and 0.682 (95% CI=0.596-0.768) for the primary dataset. The three-year and five-year OS probabilities generated by the nomogram were plotted against the observed outcomes, as shown in Figure 6B–6E. The probabilities determined by the nomogram closely approximated the actual probabilities, especially in the entire dataset.

**Figure 7:**
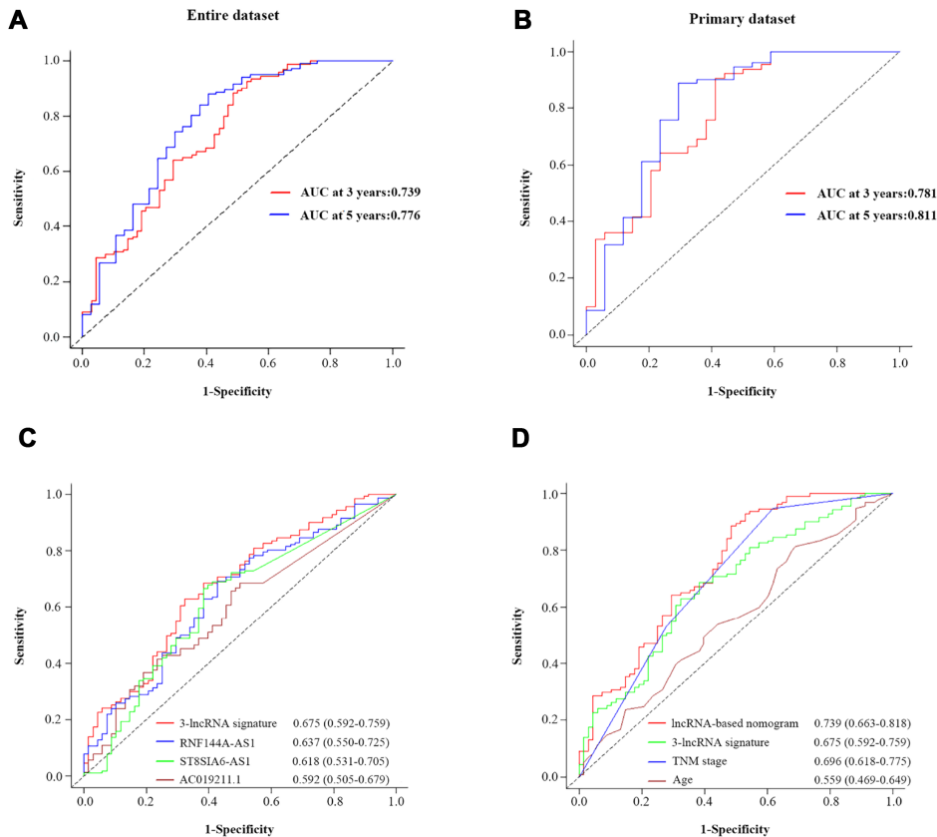
**The prognostic value of the composite nomogram in comparison with other prognostic factors.** Time-dependent ROC curves of the nomogram for predicting OS in the entire dataset (**A**) and the primary dataset (**B**). (**C**) The prognostic accuracy of the three-lncRNA signature compared with those of single lncRNAs. (**D**) The prognostic accuracy of the three-lncRNA-based prognostic nomogram compared with those of the three-lncRNA signature, TNM stage and age.

We further assessed the prognostic performance of the nomogram in a time-dependent ROC curve analysis. The AUC of the nomogram was 0.739 (95% CI=0.661-0.818) at three years and 0.779 (95% CI=0.681-0.872) at five years in the entire dataset (Figure 7A). In the primary dataset, the AUC was 0.781 (95% CI=0.679-0.883) at three years and 0.811 (95% CI=0.675-0.948) at five years (Figure 7B).

### Survival prediction power: comparison of the three-lncRNA signature-based nomogram and other clinical risk factors

To compare the predictive sensitivities and specificities of different prognostic factors, we used time-dependent ROC curves. As shown in Figure 7C, the AUCs of the individual lncRNAs at three years were 0.637 (RNF144A-AS1; 95% CI=0.550-0.725), 0.618 (ST8SIA6-AS1; 95% CI=0531-0.705) and 0.592 (ACO19211.1; 95% CI=0.505-0.679); thus, all of them were lower than that of the three-lncRNA signature (0.675, 95% CI=0.592-0.759). Although the three-lncRNA signature outperformed the individual lncRNAs, it still had a lower predictive efficiency than the TNM stage (Figure 7D). More importantly, the predictive performance of the three-lncRNA-based nomogram (AUC=0.739, 95% CI=0.663-0.818) was superior to the performance of the three-lncRNA signature (AUC=0.675, 95% CI=0.592-0.759), the TNM stage (AUC=0.696, 95% CI=0.618-0.775) and age (AUC=0.559, 95% CI=0.469-0.649). Thus, the newly developed prognostic nomogram concentrated the advantages of the three-lncRNA signature and two clinical risk factors, improving their prognostic predictive efficiency for BCa patients.

### Functional characteristics of the three-lncRNA signature

**Figure 8:**
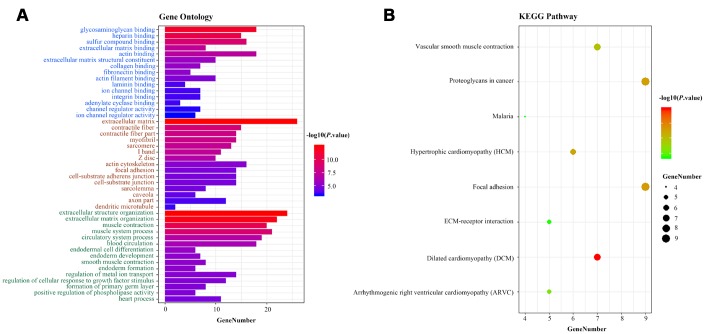
**Functional enrichment analysis of the three-lncRNA signature.** (**A**) GO enrichment analysis. Blue, brown and green words represent the GO terms for molecular functions, cellular components and biological processes, respectively. (**B**) KEGG enrichment analysis. The x-axis represents the number of genes, while the y-axis displays the GO terms and KEGG pathways. The color represents the *P* value.

To deduce the potential function of the three-lncRNA signature in BCa tumorigenesis and development, we performed a functional enrichment analysis of Gene Ontology (GO) terms and Kyoto Encyclopedia of Gene and Genomes (KEGG) pathways for mRNAs that were co-expressed with the OS-related lncRNAs in the 414 BCa samples. The levels of 184 DEMs correlated positively with the levels of at least one of the three OS-related lncRNAs (Pearson correlation coefficient >0.30). A GO enrichment analysis indicated that these co-expressed DEMs were significantly involved in 196 GO terms, including 114 terms in biological processes, 32 terms in cellular components and 17 terms in molecular functions (Supplementary Table 3). These GO terms were primarily enriched in glycosaminoglycan binding, extracellular matrix binding and extracellular structure organization (Figure 8A). Similar results were found in the KEGG pathway enrichment analysis (Figure 8B). Thus, the three-lncRNA signature mostly influenced the extracellular matrix, possibly altering cellular activities such as adhesion and migration.

### RNF144A-AS1, one of the three OS-related lncRNAs, promoted BCa cell migration and invasion *in vitro*

**Figure 9:**
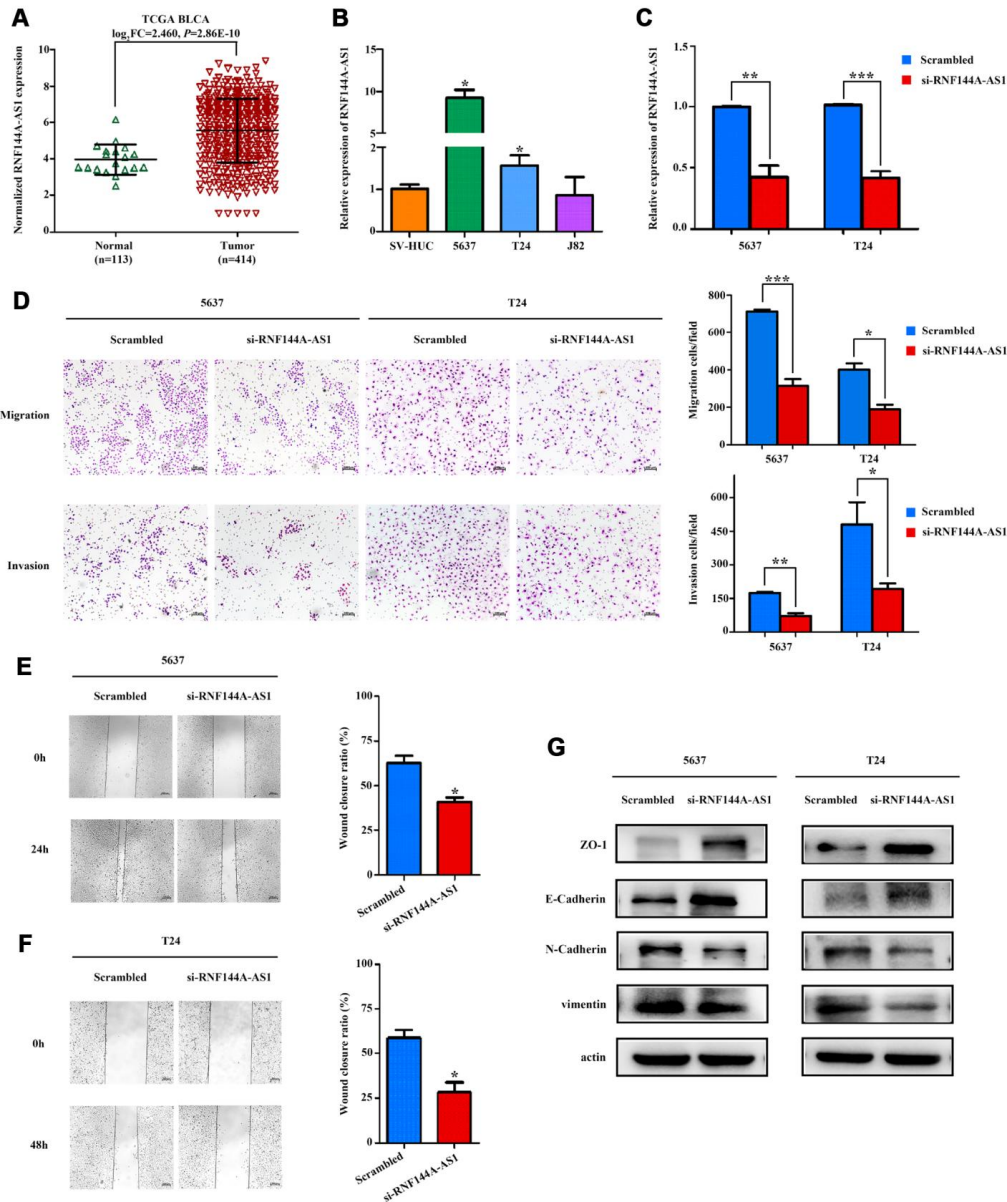
**RNF144A-AS1 enhances the invasion and migration of bladder cancer cells *in vitro.*** (**A**) The expression of RNF144A-AS1 in samples from TCGA-BLCA Project. (**B**) Quantitative real-time PCR analysis of RNF144A-AS1 expression in 5637, T24, J82 and SV-HUC cells. (**C**) Quantitative real-time PCR analysis of RNF144A-AS1 expression in RNF144A-AS1-silenced cells and scrambled-siRNA-treated cells. (**D**) The migration and invasion abilities of 5637 and T24 cells were assessed with Transwell assays after the knockdown of RNF144A-AS1. (Left panel) Representative images of migration (upper) and invasion (lower) assays. (Right panel) The number of cells that migrated or invaded are shown in the histogram. The effects of knocking down RNF144A-AS1 on the migration of 5637 (**E**) and T24 cells (**F**) were assessed with wound-healing assays. Representative images (left panel) and histogram (right panel). (**G**) The protein levels of E-cadherin, ZO-1, N-cadherin and Vimentin were detected by Western blotting in the RNF144A-AS1-knockdown group. Data are represented as the mean ± standard deviation of triplicate determinations from three independent experiments. Statistical significance was assessed with an unpaired Student’s t test (two-tailed test). *P<0.05, **P<0.01 and ***P<0.001.

We next evaluated whether these OS-related lncRNAs promoted the development of BCa. After examining the fold-changes of the three OS-related lncRNAs and the number of DEMs co-expressed with them (Supplementary Table 4), we selected RNF144A-AS1 for further functional assays. We then detected the expression of RNF144A-AS1 in 27 BCa tissues and 27 normal bladder tissues. Consistent with the expression profiles from TCGA-BLCA Project (Figure 9A), RNF144A-AS1 expression was greater in BCa tissues than in normal bladder tissues (Supplementary Figure 3). We next measured the baseline levels of RNA144A-AS1 in a panel of BCa cell lines (5637, T24 and J82) and a normal uroepithelial cell line (SV-HUC). RNA144A-AS1 expression was significantly greater in 5637 and T24 cells than in SV-HUC cells (Figure 9B).

Subsequently, we transfected RNF144A-AS1 pooled siRNA into 5637 and T24 cells. A quantitative real-time PCR analysis revealed that RNF144A-AS1 was significantly downregulated in 5637 and T24 cells after transfection (Figure 9C). Notably, Transwell and wound-healing assays demonstrated that the knockdown of RNF144A-AS1 dramatically attenuated the migratory and invasive abilities of 5637 and T24 cells (Figure 9D–9F).

The epithelial-mesenchymal transition (EMT) is a critical process during tumor invasion and metastasis. To further investigate the involvement of RNA144A-AS1 in the molecular pathological course of BCa, we measured the protein expression of EMT markers in RNA144A-AS1-siRNA-treated BCa cells. After the knockdown of RNF144A-AS1, the expression of epithelial markers (E-cadherin and ZO-1) increased, while the expression of mesenchymal markers (N-cadherin and Vimentin) decreased in BCa cells (Figure 9G). These results indicated that RNF144A-AS1 promoted the EMT and likely enhanced the migration and invasion of BCa cells.

## DISCUSSION

Currently, prognostic predictions for BCa patients largely rely on the American Joint Committee on Cancer TNM staging system [11, 34, 35]. However, the TNM system is constrained by the assumption that there is a blunt correlation between anatomical disease progression and stage progression. In fact, patients with similar anatomical spread can exhibit variable responses to therapy and a wide range of outcomes. A series of genomic landscape discoveries have demonstrated that this phenomenon may be due to tumor heterogeneity, which partly arises from genomic heterogeneity [36–38]. Forcing such patients into the same stage can introduce heterogeneity into clinical decision-making. Therefore, a reliable prognostic model for BCa is urgently needed in the era of precision medicine.

LncRNAs have been found to regulate almost every cellular process, and their own expression patterns seem to be rigorously regulated both under physiological conditions and in several disease states, including cancer [21, 39, 40]. In the present study, based on public high-throughput lncRNA expression profiles and clinical data from TCGA-BLCA Project, we discovered a novel three-lncRNA signature that could effectively identify high-risk BCa patients. These high-risk patients exhibited significantly shorter survival than those in the low-risk group.

As interest in personalized medicine has grown, a number of prognostic risk classifiers have been identified and found to enhance survival predictions in a variety of cancers [41–48]. However, most of these studies have focused only on statistical power in the screening of molecular markers, without regard for their clinical significance. Recent studies have indicated that, in addition to the TNM stage, age is also a simple but useful predictor of survival in BCa [49]. In the present study, we combined the traditional wisdom of these clinical factors with molecular profiling. Ultimately, we constructed a three-lncRNA signature-based nomogram to quantify an individual’s probability of OS. The predictive performance of our proposed prognostic nomogram was superior to those of the three-lncRNA signature, the traditional TNM stage or age alone. This objective probability scale should be simple for patients and clinicians to understand and use in clinical practice [50].

One advantage of our nomogram is its simplicity. Prognostic models are designed to identify the associations between risk factors and outcomes based on essential features, and should be accurate and parsimonious [51]. Our three-lncRNA signature-based nomogram relies on routinely available variables, including genetic differences (the three-lncRNA signature), a histopathological characteristic (TNM stage) and a baseline demographic factor (age). Thus, clinicians can easily estimate outcomes and make decisions for individual BCa patients.

The most attractive biomarkers for clinical applications are those that provide accurate prognoses for patients, stratify patients into different risk groups and thus help clinicians choose the most effective treatment. In this study, the predictive capacity of our three-lncRNA signature was independent from those of conventional clinical factors including age, TNM stage, lymph node metastasis and distant metastasis. In our stratified analysis, the three-lncRNA signature performed well for risk stratification in the stage-II, stage-III and ≥65-year-old subgroups. Notably, however, its classification efficiency was limited in the stage-IV and <65-year-old subgroups.

Although a large number of lncRNAs have been reported, few of them have been characterized for their function and mechanism. The functional expression patterns of lncRNAs tend to correlate with their highly specific transcript abundance [52–54]. In the present study, we inferred the potential functions of the three OS-related lncRNAs (RNF144A-AS1, AC019211.1 and ST8SIAS-AS1) based on a functional assessment of their co-expressed DEMs, as described in previous studies [45, 46, 55]. GO and KEGG enrichment analyses revealed that the co-expressed DEMs were primarily enriched in the extracellular matrix binding and extracellular matrix organization, which are involved in the development of BCa.

We performed further functional assays on RNF144A-AS1, one of the three OS-related lncRNAs. Transwell and wound-healing assays demonstrated that knocking down RNF144A-AS1 impaired the invasion and migration abilities of BCa cells. Knocking down RNF144A-AS1 also significantly inhibited the EMT, a key contributor to tumor invasion and metastasis, by inducing the expression of epithelial markers (E-cadherin and ZO-1) and suppressing the expression of mesenchymal markers (N-cadherin and Vimentin). Thus, silencing RNF144A-AS1 in BCa cells may prevent the EMT, thereby reducing tumor motility and invasiveness.

Although our newly proposed prognostic nomogram performed well in predicting survival for BCa patients, this study still had several limitations. Firstly, the database of TCGA lacks certain important pre- and postoperative parameters (*e.g.,* chemotherapy, radiotherapy, immunotherapy), so we could not carry out a comprehensive survival analysis with these potential factors. Secondly, we validated our prognostic model by simply applying it to the dataset originating from TCGA-BLCA Project. To reduce the risk of overfitting, we searched for independent cohorts in the Gene Expression Omnibus and Oncomine databases. Unfortunately, due to the limited number of BCa patients and clinical prognostic details, we could not find a cohort that met our validation requirements. We are actively gathering samples and corresponding clinical data from a large number of BCa patients to further validate our prognostic model. Thirdly, we used data from an open-access published database, so our study design was retrospective. Therefore, prospective clinical studies are needed to validate our findings and to determine whether our nomogram improves patients’ satisfaction and outcomes.

In conclusion, we determined the altered lncRNA expression patterns of BCa patients and identified a three-lncRNA signature that could efficiently divide patients into different risk groups. Importantly, by combining this signature with conventional clinical risk factors (TNM stage and age), we developed a three-lncRNA signature-based nomogram that could accurately predict the three-year and five-year OS of BCa patients. The prognostic performance of the nomogram was superior to those of the three-lncRNA signature, the conventional TNM stage or age. Furthermore, we functionally explored one member of the three-lncRNA signature, and found that it promoted the metastasis of BCa by inducing the EMT. Therefore, we have provided a reliable, user-friendly prognostic nomogram to aid in the individualized management of BCa patients.

## MATERIALS AND METHODS

### Data source and pre-processing

The raw counts of the RNA expression profiles and the clinical data for 414 BCa patients and 19 normal control patients from the publicly available TCGA-BLCA Project were downloaded directly from the Genomic Data Commons Data Portal (https://portal.gdc.cancer.gov, updated until August 30, 2018). All expression profiles were obtained as HT-seq raw read counts and were annotated with the Ensemble reference database (ftp://ftp.ensemble.org/pub/release-93/gtf/homo_sapiens). The RNA expression profiles were normalized and variance stabilizing transformation was performed with the “DESeq2” package in R software. The present study was conducted in accordance with the publication guidelines and data access policies of TCGA (http://cancergenome.nih.gov/publications/publicationguidelines).

### Screening of differentially expressed RNAs

DELs and DEMs between BCa samples and normal control samples were detected with the “DESeq2” package in R software. We defined lncRNAs with adjusted *P* values <0.01 and log_2_|fold change| values >2 as DELs. DEMs were defined in the same manner. Volcano plots and heatmaps were visualized with the “ggplot2” and “pheatmap” packages of R software, respectively.

### Identification of OS-related lncRNAs in BCa patients

To identify prognostic lncRNAs, we removed patients without accurate survival data, such as survival for less than 0 days. The association between DEL expression and OS was evaluated by univariate CPHR analysis and the Kaplan-Meier method. Only DELs with *P* values <0.05 and with logical consistency between their expression and prognostic effects were considered as candidate OS-related lncRNAs. After excluding patients without defined clinical characteristics, we obtained 376 BCa patients (the ‘entire dataset’), and randomly assigned 188 of them as the ‘primary dataset’. Importantly, there were no significant differences in clinical characteristics between the two datasets. The clinical features of the BCa patients are summarized in Table 1. In the primary dataset, the candidate OS-related lncRNAs were selected for multivariate CPHR analysis (stepwise model) by SPSS software. To optimize the fitting accuracy comprehensively with a moderate amount of parameters, we computed the AIC and used it to estimate the relative quality of the statistical models for the given set of data. The best-fit predictive model with the lowest AIC was chosen.

### Identification and assessment of the three-lncRNA signature

After choosing the best-fit OS-related lncRNAs through the above steps, we performed a multivariate CPHR analysis to calculate the coefficient of each lncRNA in the primary dataset. We thereby constructed a risk score formula, weighted by the linear combination of the expression values of the best-fit OS-related lncRNAs and their corresponding estimated regression coefficients. The risk score formula was constructed as follows:

$$\text{Risk Score}=\overset{\text{n}}{\underset{\text{i}=1}{\sum }}\left(C_{i}\times Exp_{i}\right)$$

where n is the number three, *Exp_i_* is the expression value of each of the three lncRNAs and *C_i_* is the corresponding estimated regression coefficient from the multivariate CPHR analysis. Using the median risk score from the primary dataset as the cut-off value, we divided patients in both the primary dataset and the entire dataset into high-risk and low-risk groups. The Kaplan-Meier method and log-rank test were performed to assess the survival differences between the high-risk and low-risk groups in each dataset. Additionally, a stratified analysis was conducted to assess whether the association of the three-lncRNA signature with OS was independent of the TNM stage and other clinical risk factors. To further evaluate the prognostic performance of the lncRNA-based classifier, we plotted time-dependent ROC curves and calculated the AUC values in each dataset, with three and five years as the defining points.

### Development of the lncRNA signature-based prognostic nomogram

To identify independent predictors of OS, we tested conventional clinical risk factors and the lncRNA-based signature through univariate and multivariate CPHR analyses of the 376 BCa patients. A prognostic nomogram was then established with the “rms” package. The abilities of the nomogram were assessed with a C-index and calibration curves to compare non-events and events or the model-predicted and actual probabilities of OS. A bootstrap validation with 1000 resamplings was used for these activities. As for the predictive performance, we also measured the AUC values based on time-dependent ROC curves.

### Function and pathway enrichment analyses

The co-expression of the three OS-related lncRNAs and the DEMs was assessed with a Pearson correlation test. To reduce false positives, we only selected co-expressed OS-related lncRNA/DEM pairs for further enrichment analysis when a positive correlation coefficient >0.3 was obtained. The “clusterProfiler” package in R was used to classify genes based on their projection at a specific level of GO terms or KEGG pathways. Functional enrichment analyses were carried out for GO terms and KEGG pathways through a hypergeometric distribution with a significance threshold of *P*<0.05.

### Human patient specimens

In total, 27 BCa tissues and 27 normal bladder tissues were obtained from patients or healthy subjects who had undergone surgery and had not received radiotherapy or chemotherapy prior to surgery at The Second Hospital of Shandong University between 2017 and 2019. None of the patients had other tumorous diseases at the time of sample collection. All samples were pathologically confirmed as BCa according to the 7th edition of the American Joint Committee on Cancer staging manual. This study was approved by the ethics committee of The Second Hospital of Shandong University.

### Cell culture and siRNA transfection

The human normal uroepithelial cell line SV-HUC and bladder cancer cell lines T24, 5637 and J82 were purchased from the Cell Bank of the Chinese Academy of Sciences (Shanghai, China). T24 and 5637 cells were cultured in RPMI-1640 medium (Gibco, Shanghai, China), while J82 and SV-HUC cells were cultured in minimum essential medium and F-12K medium (Macgene, Beijing, China), respectively. All media were supplemented with 10% fetal bovine serum (FBS; Sagecreation, Beijing, China) and 1% penicillin and streptomycin (Solarbio, Beijing, China). Cells were grown at 37 in an atmosphere of 5% CO_2_, and were tested without mycoplasma.

RNF144A-AS1 siRNA and negative control siRNA oligonucleotides were designed and synthesized by GenePharma (Shanghai, China); the sequences are listed in Supplementary Table 5. The siRNA transfections were performed with 100 nM pooled siRNA and Lipofectamine 2000 (Life Technologies) in accordance with the manufacturer’s instructions.

### RNA extraction and quantitative real-time PCR

Total RNA was extracted from cells with RNA fast 2000 Reagent (Fastagen, Shanghai, China) and quantified with a NanoDrop spectrophotometer (Thermo Fisher Scientific, Waltham, MA, USA). Then, 1 μg of total RNA was reverse-transcribed with a PrimeScript^TM^ RT Reagent Kit (Takara, Dalian, China) in a 20-μL reaction according to the manufacturer’s instructions. Quantitative real-time PCR was performed with TB Green^TM^ Premix Ex Taq^TM^ (Takara) in a 25-μL reaction containing 2 μL of cDNA, and was run on a CFX-96 real-time PCR System (Bio-Rad, Shanghai, China). The PCR primer sequences were: RNF144A-AS1 forward: 5′-CACACAGCAAGCTAGGA-3′, reverse: 5′-ACTTTCCTTGCGAGGGTTGG-3′; *GAPDH* forward: 5′-ACCCACTCCTCCACCTTTG AC-3′, reverse: 5′-TGTTGCTGTAGCCAAATTCGTT-3′. After being briefly mixed, the reaction mixture was incubated at 95°C for 30 seconds, followed by 42 cycles at 95°C for 5 seconds and 61°C for 30 seconds. All reactions were performed in triplicate, and no-template controls were included in each run. *GAPDH* was used as an endogenous control to standardize the expression of each target gene, and the 2^-ΔΔCT^ method was adopted to determine the relative target gene level.

### Transwell assay

The Transwell assay was performed with a 24-well Transwell plate (8-μm pore size; Corning). After being transfected with pooled RNF144A-AS1 siRNA or control siRNA, 5×10^4^ T24 cells or 8×10^4^ 5637 cells in 200 μL of serum-free medium were seeded into the upper chamber, while the lower chamber was filled with 800 μL of medium supplemented with 20% FBS. After 24 hours, the chamber was washed with phosphate-buffered saline (PBS). Then, the non-migrating cells in the upper chamber were removed with a cotton swab, while the cells that had migrated to the lower surface were fixed in methanol, stained with Giemsa and photographed under a microscope (Zeiss, Axio Observer). The images were processed with ImageJ Pro Plus (version 6.0). The invasion assays were performed by a similar method, except that the upper surface of the chamber was pre-coated with Matrigel (BD Biosciences) and the number of cells was doubled.

### Wound-healing assay

Cells that had been transfected with pooled RNF144A-AS1 siRNA or control siRNA were seeded into 12-well plates to form a confluent monolayer. An artificial homogenous wound was produced with a sterile 200-μL pipet tip (T-200-Y, Axygen), and the well was carefully washed with PBS to remove cell debris. Then, the cells were cultured in medium supplemented with 2% FBS. Images were taken at 0, 24 and 48 hours with an inverted microscope (Zeiss, Axio Observer), and were analyzed with ImageJ Pro Plus (version 6.0).

### Western blotting

Cells were washed with PBS and lysed with a radioimmunoprecipitation assay lysis buffer containing a protease inhibitor. The proteins were quantified with a bicinchoninic acid protein assay kit. Then, 40 μg of total protein was electrophoretically separated on a 6% or 10% sodium dodecyl sulfate polyacrylamide gel and blotted onto a polyvinylidene difluoride membrane (Millipore, USA). The membrane was blocked with 5% bovine serum albumin for 1 hour, and then was incubated with the primary antibody (at a 1:1000 dilution) against β-actin, E-cadherin, N-cadherin and ZO-1 (Cell Signaling Technology, USA) or Vimentin (Abcam, USA) overnight at 4°C. After being washed three times with Tris-buffered saline-Tween, the membrane was incubated with a secondary antibody (at a 1:5000 dilution) at room temperature for 1 hour. After another three washes, the bands were visualized with an enhanced chemiluminescence system (Bio-Rad Laboratories). β-actin was used as an internal control.

### Statistical analysis

The χ2 test was used to compare the associations of continuous and categorical variables between the primary dataset and the entire dataset. Univariate CPHR analysis and the Kaplan-Meier method were used to obtain candidate OS-related lncRNAs. Multivariate CPHR analysis (stepwise model) was then performed to screen variables and determine the risk score formula. For survival analysis, the Kaplan-Meier method was used to plot survival curves, which were compared through log-rank tests. A time-dependent ROC curve was used to assess the specificity and sensitivity of the prognostic prediction at each time point. The nomogram incorporating both the lncRNA signature and independent clinical risk factors was developed through a multivariate CPHR analysis and was validated with the C-index and calibration curves. For the functional assays *in vitro*, all quantitative data are presented as the mean ± standard deviation of three independent experiments. Differences between two groups were analyzed with Student’s t test (two-tailed test). Statistical analyses were performed with R software (version 3.5.2), SPSS software (version 23.0) or GraphPad Prism 5.0 (GraphPad, La Jolla, CA, USA). A *P* value <0.05 was considered statistically significant unless otherwise indicated.

## Supplementary Material

Supplementary Figures

Supplementary Tables

Supplementary Table 3
