# Supplementary material for: A nomogram combining long non-coding RNA expression profiles and clinical factors predicts survival in patients with bladder cancer
Source: Aging (Albany NY). 2020 Feb 12;12(3):2857–79. doi: 10.18632/aging.102782 (PMC7041749; doi:10.18632/aging.102782)
Supplement: Supplementary Figures [file aging-12-102782-s003..pdf]

# SUPPLEMENTARY FIGURES

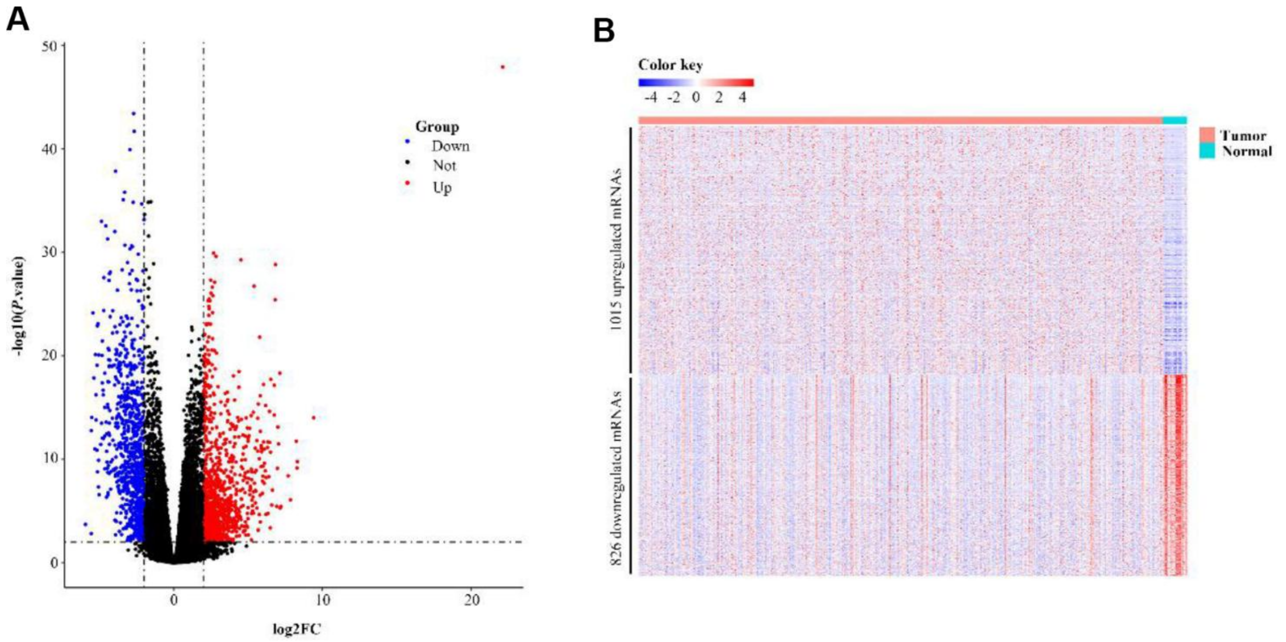


**Supplementary Figure 1. Volcano plot and heatmap of 1841 mRNAs in bladder cancer patients from TCGA-BLCA Project.** (**A**) Volcano plot of 1841 mRNAs in bladder cancer samples from TCGA-BLCA Project. (**B**) Heatmap of 1841 mRNAs in bladder cancer samples from TCGA-BLCA Project. Blue indicates downregulated mRNAs, and red represents upregulated mRNAs.

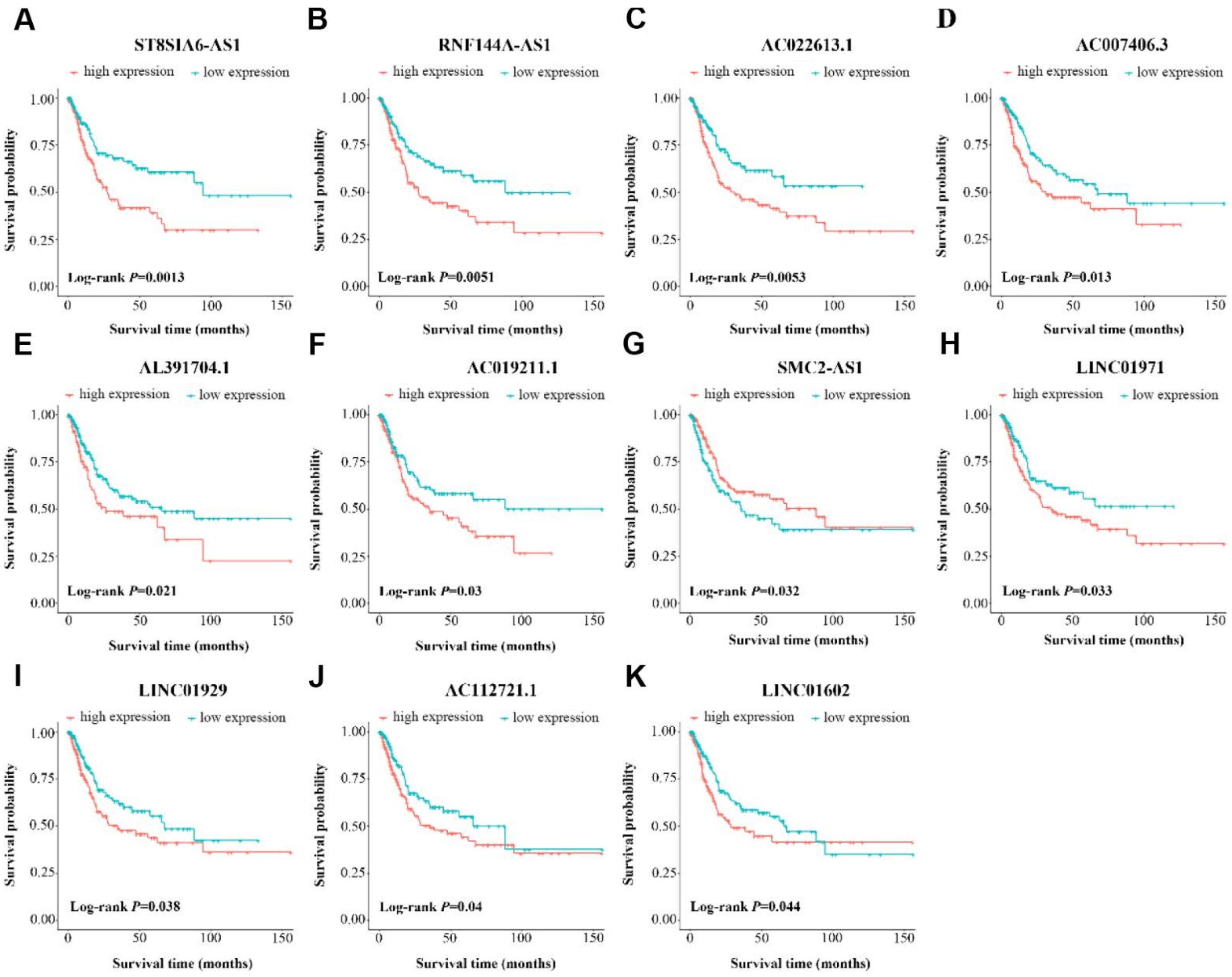


**Supplementary Figure 2. Kaplan-Meier curves of OS for 410 bladder cancer patients based on the expression of candidate OS-related lncRNAs.** (**A**) ST8SIA6-AS1. (**B**) NF144A-AS1. (**C**) AC022613.1. (**D**) AC007406.3. (**E**) AL391704.1. (**F**) AC019211.1. (**G**) SMC2-AS1. (**H**) LINC01971. (**I**) LINC01929. (**J**) AC112721.1. (**K**) LINC01602.

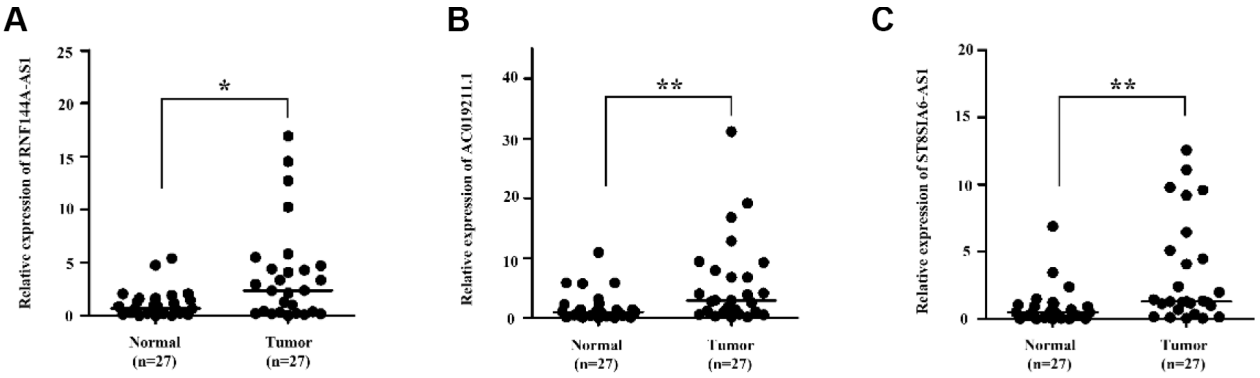


**Supplementary Figure 3.** The expression of RNF144A-AS1 (**A**), ACO19211.1 (**B**) and ST8SIA6-AS1 (**C**) in 27 bladder cancer tissues and 27 normal bladder tissues.
