# Supplementary material for: A nomogram combining long non-coding RNA expression profiles and clinical factors predicts survival in patients with bladder cancer
Source: Aging (Albany NY). 2020 Feb 12;12(3):2857–79. doi: 10.18632/aging.102782 (PMC7041749; doi:10.18632/aging.102782)
Supplement: Supplementary Tables [file aging-12-102782-s002..pdf]

SUPPLEMENTARY TABLES

Supplementary Table 1. Candidate lncRNAs significantly associated with the OS of 410 bladder cancer patients.

Gene name	log rank *P* value	Cox *P* value	HR	95%CI	
ST8SIA6-AS1	1.27E-03	1.52E-03	1.868424	1.269702	2.749471
RNF144A-AS1	5.09E-03	5.62E-03	1.715524	1.170852	2.513573
AC022613.1	5.34E-03	5.95E-03	1.721833	1.169022	2.536059
AC007406.3	1.28E-02	1.36E-02	1.607132	1.102546	2.342646
AL391704.1	0.021288	0.022361	1.560537	1.065205	2.286205
AC019211.1	0.030055	0.031251	1.509522	1.037767	2.195731
SMC2-AS1	0.031609	0.032703	0.666044	0.4587	0.967111
LINC01971	0.033246	0.034566	1.509195	1.030332	2.210616
LINC01929	0.037970	0.039302	1.488602	1.019696	2.173132
AC112721.1	0.040431	0.041807	1.484519	1.014757	2.171747
LINC01602	0.043954	0.045246	1.463558	1.008097	2.124797

Abbreviations: HR, hazard ratio; CI, confidence interval.

Supplementary Table 2 Univariate and multivariate CPHR analyses of the three-lncRNA signature and clinical risk factors in the primary dataset.

Characteristic	Univariate analysis		Multivariate analysis	
	HR (95%CI)	P value	HR (95%CI)	P value
Age (≥65 *vs.* <65)	1.167 (0.653-2.085)	0.603		
Gender (male/female)	0.827 (0.472-1.447)	0.505		
TNM stage (III-IV *vs.* I-II)	4.039 (1.608-10.140)	**0.003**	3.436 (1.360-8.682)	**0.009**
Tumor stage (T3-T4 *vs.* T0-T2)	2.770 (1.353-5.670)	**0.005**		
Lymph node metastasis (yes *vs.* no)	3.040 (1.724-5.361)	**<0.001**		
Distant metastasis (yes *vs.* no)	2.192 (0.510-9.419)	0.291		
Risk score (high *vs.* low)	2.698 (1.539-4.729)	**<0.001**	2.368 (1.345-4.168)	**0.003**

Note: Bold values indicate statistical significance (P<0.05).
Abbreviations: HR, hazard ratio; CI, confidence interval.

Please browse Full Text version to see the data of Supplementary Table 3

Supplementary Table 3. Functional enrichment analysis of GO terms for DEMs that were positively co-expressed with OS-related lncRNAs.

Supplementary Table 4. Summary of the three OS-related lncRNAs.

Gene name	Down/up-regulated	log2FoldChange	P value	co-expressed mRNAs
RNF144A-AS1	Up	2.460	2.86E-10	175/184
AC019211.1	Up	2.577	7.43E-05	4/184
ST8SIA6-AS1	Up	4.073	1.23E-05	5/184

Abbreviations: OS, overall survival.

Supplementary Table 5. The siRNA oligonucleotides against RNF144A-AS1 and the negative control.

siRNA	The siRNA oligonucleotides
RNF144A-AS1-si#1	Sense: GCCAAGAAAUGGCAAAGAUTT<br>Antisense: AUCUUUGGCAUUUCUUGGCTT
RNF144A-AS1-si#2	Sense: CCAUGUGAACUGAAGUCAATT<br>Antisense: UUGACUUCAGUUCACAUGGGTT
RNF144A-AS1-si#3	Sense: GCAGACAGCACAAGACUUUTT<br>Antisense: AAAGUCUUGUGCUGUCUGCTT
Negative control	Sense: UUCUCCGAACGUGUCACGUTT<br>Antisense: ACGUGACACGUUCGGAGAATT
