# Supplementary material for: A nomogram combining long non-coding RNA expression profiles and clinical factors predicts survival in patients with bladder cancer
Source: Aging (Albany NY). 2020 Feb 12;12(3):2857–79. doi: 10.18632/aging.102782 (PMC7041749; doi:10.18632/aging.102782)
Supplement: Supplementary Table 3 [file aging-12-102782-s001..docx]

**Supplementary Table 3** Functional enrichment analysis of GO terms for positively co-expressed DEMs with OS-related lncRNAs.

| **ID** | **Function Description** | ***P* value** | **GeneRatio** | **BgRatio** |
| --- | --- | --- | --- | --- |
| GO:0043062 | extracellular structure organization | 3.42E-13 | 24/170 | 402/18493 |
| GO:0030198 | extracellular matrix organization | 1.14E-12 | 22/170 | 348/18493 |
| GO:0006936 | muscle contraction | 8.45E-11 | 20/170 | 351/18493 |
| GO:0003012 | muscle system process | 1.36E-09 | 21/170 | 456/18493 |
| GO:0003013 | circulatory system process | 1.82E-07 | 19/170 | 500/18493 |
| GO:0008015 | blood circulation | 6.79E-07 | 18/170 | 492/18493 |
| GO:0035987 | endodermal cell differentiation | 3.34E-06 | 6/170 | 45/18493 |
| GO:0007492 | endoderm development | 5.26E-06 | 7/170 | 74/18493 |
| GO:0006939 | smooth muscle contraction | 5.65E-06 | 8/170 | 105/18493 |
| GO:0001706 | endoderm formation | 6.28E-06 | 6/170 | 50/18493 |
| GO:0010959 | regulation of metal ion transport | 1.03E-05 | 14/170 | 376/18493 |
| GO:0090287 | regulation of cellular response to growth factor stimulus | 1.21E-05 | 12/170 | 282/18493 |
| GO:0001704 | formation of primary germ layer | 1.34E-05 | 8/170 | 118/18493 |
| GO:0010518 | positive regulation of phospholipase activity | 1.51E-05 | 6/170 | 58/18493 |
| GO:0003015 | heart process | 2.22E-05 | 11/170 | 252/18493 |
| GO:0007517 | muscle organ development | 2.22E-05 | 14/170 | 403/18493 |
| GO:0006816 | calcium ion transport | 2.91E-05 | 14/170 | 413/18493 |
| GO:0045444 | fat cell differentiation | 3.34E-05 | 10/170 | 217/18493 |
| GO:0086003 | cardiac muscle cell contraction | 3.47E-05 | 6/170 | 67/18493 |
| GO:0060485 | mesenchyme development | 4.04E-05 | 11/170 | 269/18493 |
| GO:0010517 | regulation of phospholipase activity | 4.10E-05 | 6/170 | 69/18493 |
| GO:0060193 | positive regulation of lipase activity | 4.45E-05 | 6/170 | 70/18493 |
| GO:0007369 | gastrulation | 4.61E-05 | 9/170 | 181/18493 |
| GO:0014829 | vascular smooth muscle contraction | 5.32E-05 | 4/170 | 23/18493 |
| GO:0002040 | sprouting angiogenesis | 6.45E-05 | 9/170 | 189/18493 |
| GO:0031589 | cell-substrate adhesion | 7.08E-05 | 12/170 | 338/18493 |
| GO:0045599 | negative regulation of fat cell differentiation | 8.51E-05 | 5/170 | 49/18493 |
| GO:0014820 | tonic smooth muscle contraction | 8.73E-05 | 3/170 | 10/18493 |
| GO:0070252 | actin-mediated cell contraction | 8.84E-05 | 7/170 | 114/18493 |
| GO:0060047 | heart contraction | 8.93E-05 | 10/170 | 244/18493 |
| GO:0070838 | divalent metal ion transport | 9.07E-05 | 14/170 | 459/18493 |
| GO:0072511 | divalent inorganic cation transport | 9.72E-05 | 14/170 | 462/18493 |
| GO:0090130 | tissue migration | 1.13E-04 | 12/170 | 355/18493 |
| GO:0051216 | cartilage development | 1.25E-04 | 9/170 | 206/18493 |
| GO:0034394 | protein localization to cell surface | 1.36E-04 | 5/170 | 54/18493 |
| GO:0002009 | morphogenesis of an epithelium | 1.42E-04 | 14/170 | 479/18493 |
| GO:1903522 | regulation of blood circulation | 1.60E-04 | 10/170 | 262/18493 |
| GO:0061448 | connective tissue development | 1.65E-04 | 10/170 | 263/18493 |
| GO:0099623 | regulation of cardiac muscle cell membrane repolarization | 1.79E-04 | 4/170 | 31/18493 |
| GO:0045598 | regulation of fat cell differentiation | 1.82E-04 | 7/170 | 128/18493 |
| GO:0008016 | regulation of heart contraction | 1.84E-04 | 9/170 | 217/18493 |
| GO:0001503 | ossification | 2.12E-04 | 12/170 | 380/18493 |
| GO:0060048 | cardiac muscle contraction | 2.31E-04 | 7/170 | 133/18493 |
| GO:0060191 | regulation of lipase activity | 2.44E-04 | 6/170 | 95/18493 |
| GO:0030048 | actin filament-based movement | 2.65E-04 | 7/170 | 136/18493 |
| GO:0034765 | regulation of ion transmembrane transport | 3.00E-04 | 13/170 | 454/18493 |
| GO:0007229 | integrin-mediated signaling pathway | 3.06E-04 | 6/170 | 99/18493 |
| GO:0010632 | regulation of epithelial cell migration | 3.06E-04 | 10/170 | 284/18493 |
| GO:0001667 | ameboidal-type cell migration | 3.13E-04 | 13/170 | 456/18493 |
| GO:0007015 | actin filament organization | 3.16E-04 | 12/170 | 397/18493 |
| GO:0048705 | skeletal system morphogenesis | 3.43E-04 | 9/170 | 236/18493 |
| GO:0006813 | potassium ion transport | 3.65E-04 | 9/170 | 238/18493 |
| GO:0032412 | regulation of ion transmembrane transporter activity | 3.65E-04 | 9/170 | 238/18493 |
| GO:0051924 | regulation of calcium ion transport | 3.76E-04 | 9/170 | 239/18493 |
| GO:0035988 | chondrocyte proliferation | 3.91E-04 | 3/170 | 16/18493 |
| GO:1901841 | regulation of high voltage-gated calcium channel activity | 3.91E-04 | 3/170 | 16/18493 |
| GO:0090132 | epithelium migration | 3.95E-04 | 11/170 | 349/18493 |
| GO:0060306 | regulation of membrane repolarization | 3.99E-04 | 4/170 | 38/18493 |
| GO:0022898 | regulation of transmembrane transporter activity | 4.50E-04 | 9/170 | 245/18493 |
| GO:0060665 | regulation of branching involved in salivary gland morphogenesis by mesenchymal-epithelial signaling | 4.98E-04 | 2/170 | 4/18493 |
| GO:1904849 | positive regulation of cell chemotaxis to fibroblast growth factor | 4.98E-04 | 2/170 | 4/18493 |
| GO:2000546 | positive regulation of endothelial cell chemotaxis to fibroblast growth factor | 4.98E-04 | 2/170 | 4/18493 |
| GO:0099622 | cardiac muscle cell membrane repolarization | 5.35E-04 | 4/170 | 41/18493 |
| GO:0090257 | regulation of muscle system process | 5.52E-04 | 9/170 | 252/18493 |
| GO:0010863 | positive regulation of phospholipase C activity | 5.88E-04 | 4/170 | 42/18493 |
| GO:0086004 | regulation of cardiac muscle cell contraction | 5.88E-04 | 4/170 | 42/18493 |
| GO:0022617 | extracellular matrix disassembly | 5.98E-04 | 5/170 | 74/18493 |
| GO:0003416 | endochondral bone growth | 6.43E-04 | 4/170 | 43/18493 |
| GO:1904062 | regulation of cation transmembrane transport | 6.70E-04 | 10/170 | 314/18493 |
| GO:0032409 | regulation of transporter activity | 6.90E-04 | 9/170 | 260/18493 |
| GO:1900274 | regulation of phospholipase C activity | 7.02E-04 | 4/170 | 44/18493 |
| GO:0051928 | positive regulation of calcium ion transport | 7.12E-04 | 6/170 | 116/18493 |
| GO:0003018 | vascular process in circulatory system | 7.30E-04 | 7/170 | 161/18493 |
| GO:0032970 | regulation of actin filament-based process | 7.34E-04 | 11/170 | 376/18493 |
| GO:0002062 | chondrocyte differentiation | 7.45E-04 | 6/170 | 117/18493 |
| GO:2001257 | regulation of cation channel activity | 7.58E-04 | 7/170 | 162/18493 |
| GO:0010463 | mesenchymal cell proliferation | 7.66E-04 | 4/170 | 45/18493 |
| GO:0098868 | bone growth | 7.66E-04 | 4/170 | 45/18493 |
| GO:0010811 | positive regulation of cell-substrate adhesion | 7.79E-04 | 6/170 | 118/18493 |
| GO:0055117 | regulation of cardiac muscle contraction | 8.06E-04 | 5/170 | 79/18493 |
| GO:0006937 | regulation of muscle contraction | 8.14E-04 | 7/170 | 164/18493 |
| GO:0010634 | positive regulation of epithelial cell migration | 8.44E-04 | 7/170 | 165/18493 |
| GO:0071621 | granulocyte chemotaxis | 8.50E-04 | 6/170 | 120/18493 |
| GO:1903115 | regulation of actin filament-based movement | 9.03E-04 | 4/170 | 47/18493 |
| GO:0006941 | striated muscle contraction | 9.38E-04 | 7/170 | 168/18493 |
| GO:0030324 | lung development | 9.71E-04 | 7/170 | 169/18493 |
| GO:1900746 | regulation of vascular endothelial growth factor signaling pathway | 1.03E-03 | 3/170 | 22/18493 |
| GO:0010594 | regulation of endothelial cell migration | 1.06E-03 | 8/170 | 222/18493 |
| GO:0086009 | membrane repolarization | 1.06E-03 | 4/170 | 49/18493 |
| GO:0060537 | muscle tissue development | 1.10E-03 | 11/170 | 395/18493 |
| GO:0030323 | respiratory tube development | 1.11E-03 | 7/170 | 173/18493 |
| GO:1902547 | regulation of cellular response to vascular endothelial growth factor stimulus | 1.18E-03 | 3/170 | 23/18493 |
| GO:1901019 | regulation of calcium ion transmembrane transporter activity | 1.18E-03 | 5/170 | 86/18493 |
| GO:0035637 | multicellular organismal signaling | 1.23E-03 | 7/170 | 176/18493 |
| GO:0014832 | urinary bladder smooth muscle contraction | 1.23E-03 | 2/170 | 6/18493 |
| GO:0014848 | urinary tract smooth muscle contraction | 1.23E-03 | 2/170 | 6/18493 |
| GO:0021869 | forebrain ventricular zone progenitor cell division | 1.23E-03 | 2/170 | 6/18493 |
| GO:1901897 | regulation of relaxation of cardiac muscle | 1.23E-03 | 2/170 | 6/18493 |
| GO:0035296 | regulation of tube diameter | 1.24E-03 | 6/170 | 129/18493 |
| GO:0097746 | regulation of blood vessel diameter | 1.24E-03 | 6/170 | 129/18493 |
| GO:0030199 | collagen fibril organization | 1.32E-03 | 4/170 | 52/18493 |
| GO:2001026 | regulation of endothelial cell chemotaxis | 1.34E-03 | 3/170 | 24/18493 |
| GO:0010631 | epithelial cell migration | 1.40E-03 | 10/170 | 346/18493 |
| GO:0061138 | morphogenesis of a branching epithelium | 1.49E-03 | 7/170 | 182/18493 |
| GO:0097530 | granulocyte migration | 1.50E-03 | 6/170 | 134/18493 |
| GO:0090092 | regulation of transmembrane receptor protein serine/threonine kinase signaling pathway | 1.52E-03 | 8/170 | 235/18493 |
| GO:0050880 | regulation of blood vessel size | 1.62E-03 | 6/170 | 136/18493 |
| GO:0002011 | morphogenesis of an epithelial sheet | 1.63E-03 | 4/170 | 55/18493 |
| GO:0034329 | cell junction assembly | 1.64E-03 | 8/170 | 238/18493 |
| GO:0006942 | regulation of striated muscle contraction | 1.68E-03 | 5/170 | 93/18493 |
| GO:0035150 | regulation of tube size | 1.68E-03 | 6/170 | 137/18493 |
| GO:0060307 | regulation of ventricular cardiac muscle cell membrane repolarization | 1.70E-03 | 3/170 | 26/18493 |
| GO:0098910 | regulation of atrial cardiac muscle cell action potential | 1.71E-03 | 2/170 | 7/18493 |
| GO:0086002 | cardiac muscle cell action potential involved in contraction | 1.74E-03 | 4/170 | 56/18493 |
| GO:0005539 | glycosaminoglycan binding | 3.73E-12 | 18/167 | 222/17632 |
| GO:0008201 | heparin binding | 4.19E-11 | 15/167 | 163/17632 |
| GO:1901681 | sulfur compound binding | 1.28E-09 | 16/167 | 242/17632 |
| GO:0050840 | extracellular matrix binding | 5.28E-08 | 8/167 | 56/17632 |
| GO:0003779 | actin binding | 1.05E-07 | 18/167 | 421/17632 |
| GO:0005201 | extracellular matrix structural constituent | 2.24E-06 | 10/167 | 155/17632 |
| GO:0005518 | collagen binding | 3.25E-06 | 7/167 | 67/17632 |
| GO:0001968 | fibronectin binding | 4.90E-06 | 5/167 | 27/17632 |
| GO:0051015 | actin filament binding | 1.36E-05 | 10/167 | 190/17632 |
| GO:0043236 | laminin binding | 1.53E-04 | 4/167 | 29/17632 |
| GO:0044325 | ion channel binding | 1.88E-04 | 7/167 | 125/17632 |
| GO:0005178 | integrin binding | 1.98E-04 | 7/167 | 126/17632 |
| GO:0008179 | adenylate cyclase binding | 2.23E-04 | 3/167 | 13/17632 |
| GO:0016247 | channel regulator activity | 3.94E-04 | 7/167 | 141/17632 |
| GO:0099106 | ion channel regulator activity | 5.15E-04 | 6/167 | 106/17632 |
| GO:0048495 | Roundabout binding | 8.75E-04 | 2/167 | 5/17632 |
| GO:0001786 | phosphatidylserine binding | 1.70E-03 | 4/167 | 54/17632 |
| GO:0031012 | extracellular matrix | 3.77E-13 | 26/178 | 490/19659 |
| GO:0043292 | contractile fiber | 2.77E-09 | 15/178 | 229/19659 |
| GO:0044449 | contractile fiber part | 9.70E-09 | 14/178 | 214/19659 |
| GO:0030016 | myofibril | 1.23E-08 | 14/178 | 218/19659 |
| GO:0030017 | sarcomere | 3.01E-08 | 13/178 | 197/19659 |
| GO:0031674 | I band | 4.85E-08 | 11/178 | 137/19659 |
| GO:0030018 | Z disc | 2.24E-07 | 10/178 | 126/19659 |
| GO:0015629 | actin cytoskeleton | 8.66E-06 | 16/178 | 483/19659 |
| GO:0005925 | focal adhesion | 1.86E-05 | 14/178 | 402/19659 |
| GO:0005924 | cell-substrate adherens junction | 2.02E-05 | 14/178 | 405/19659 |
| GO:0030055 | cell-substrate junction | 2.25E-05 | 14/178 | 409/19659 |
| GO:0042383 | sarcolemma | 2.89E-05 | 8/178 | 133/19659 |
| GO:0005901 | caveola | 7.05E-05 | 6/178 | 77/19659 |
| GO:0033267 | axon part | 1.57E-04 | 12/178 | 373/19659 |
| GO:1901588 | dendritic microtubule | 2.43E-04 | 2/178 | 3/19659 |
| GO:0044853 | plasma membrane raft | 3.32E-04 | 6/178 | 102/19659 |
| GO:0045121 | membrane raft | 4.67E-04 | 10/178 | 304/19659 |
| GO:0098857 | membrane microdomain | 4.79E-04 | 10/178 | 305/19659 |
| GO:0098589 | membrane region | 6.31E-04 | 10/178 | 316/19659 |
| GO:0030426 | growth cone | 7.76E-04 | 7/178 | 165/19659 |
| GO:0030427 | site of polarized growth | 8.33E-04 | 7/178 | 167/19659 |
| GO:0031252 | cell leading edge | 8.82E-04 | 11/178 | 390/19659 |
| GO:0150034 | distal axon | 1.05E-03 | 9/178 | 280/19659 |
| GO:0005581 | collagen trimer | 1.17E-03 | 5/178 | 87/19659 |
| GO:0005884 | actin filament | 2.07E-03 | 5/178 | 99/19659 |
| GO:0030863 | cortical cytoskeleton | 2.36E-03 | 5/178 | 102/19659 |
| GO:0005911 | cell-cell junction | 2.38E-03 | 11/178 | 442/19659 |
| GO:0016363 | nuclear matrix | 3.03E-03 | 5/178 | 108/19659 |
| GO:0043198 | dendritic shaft | 3.86E-03 | 3/178 | 35/19659 |
| GO:0016528 | sarcoplasm | 5.02E-03 | 4/178 | 76/19659 |
| GO:0031594 | neuromuscular junction | 5.02E-03 | 4/178 | 76/19659 |
| GO:0005938 | cell cortex | 5.23E-03 | 8/178 | 292/19659 |

Abbreviations: GO, gene ontology; DEMs, differently expressed mRNAs.

Notes:

BGRatio = M/N, M = size of the geneset, N= size of all of the unique genes in the collection of genesets.

GeneRatio = k/n, k = size of the overlap of “a vector of gene id” which input with the specific geneset, n = size of the overlap of “a vector of gene id” which input with all the members of the collection of geneset.
